# Revealing the developmental dynamics in male strobilus transcriptome of *Gnetum luofuense* using nanopore sequencing technology

**DOI:** 10.1038/s41598-021-90082-0

**Published:** 2021-05-18

**Authors:** Chen Hou, Yuxin Tian, Yingli Wang, Huiming Lian, Dongcheng Liang, Shengqing Shi, Nan Deng, Boxiang He

**Affiliations:** 1 Guangdong Provincial Key Laboratory of Silviculture, Protection and Utilization, Guangdong Academy of Forestry, Guangzhou, 510520 China; 2 Guangdong Academy of Forestry, Guangshanyilu No. 233, Longdong District, Guangzhou, 510520 China; 3 Hunan Academy of Forestry, Changsha, Hunan, No. 658 Shaoshan Road, Tianxin District, Changsha, 410004 China; 4 Hunan Cili Forest Ecosystem State Research Station, Cili, Hunan, Changsha, 410004 China; 5 State Key Laboratory of Tree Genetics and Breeding, Research Institute of Forestry, Chinese Academy of Forestry, Beijing, 100091 China

**Keywords:** Flowering, Plant development, Plant morphogenesis

## Abstract

*Gnetum* is a pantropical distributed gymnosperm genus. As being dioecious, *Gnetum* species apply female and male strobili to attract and provide nutrition to insect pollinators. Due to its unique gross morphology, a *Gnetum* male strobilus receives much attention in previous taxonomic and evolutionary studies. However, underlying molecular mechanisms that control male strobilus development and pollination adaptation have not been well studied. In the present study, nine full-length transcriptomes were sequenced from three developmental stages of the *G. luofuense* male strobili using Oxford Nanopore Technologies. In addition, weighted gene co-expression network analysis (WGCNA), and RT-qPCR analysis were performed. Our results show that a total of 3138 transcription factors and 466 long non-coding RNAs (lncRNAs) were identified, and differentially expressed lncRNAs and TFs reveal a dynamic pattern during the male strobilus development. Our results show that MADS-box and Aux/IAA TFs were differentially expressed at the three developmental stages, suggesting their important roles in the regulation of male strobilus development of *G. luofuense*. Results of WGCNA analysis and annotation of differentially expressed transcripts corroborate that the male strobilus development of *G. luofuense* is closely linked to plant hormone changes, photosynthesis, pollination drop secretion and reproductive organ defense. Our results provide a valuable resource for understanding the molecular mechanisms that drive organ evolution and pollination biology in *Gnetum*.

## Introduction

**Figure 1:**
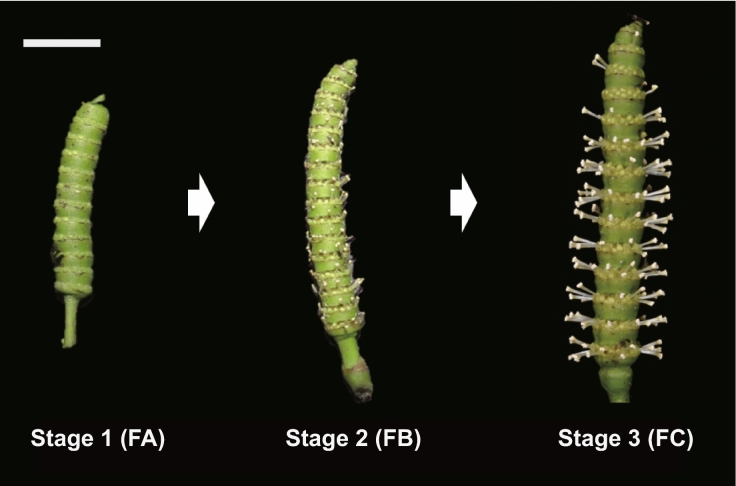
Different developmental stages of male strobili in *Gnetum luofuense* C.Y. Cheng. A white bar represents 1 cm. The photograph was made by C.H.

The genus *Gnetum* comprises approximately 40 species that are widely distributed in pantropical forests^1,2^. The leaves of *Gnetum* are edible, its stems and bark are made into string, nets, and paper, and its seeds can be used for oil and drinks^3,4^. Reproductive organs of *Gnetum* consist of female and male strobili that are composed of several layers of involucre collars^1,5^ (Fig. 1). In a male strobilus, each layer of involucre collars is composed of exposed (e.g. *G. gnemon* L.) or hidden (*G. luofuense* C.Y. Cheng) sterile ovules at the top, surrounded by several layers of male reproductive units with attached microsporangia^6^. With regard to the hidden and exposed sterile ovules, genus *Gnetum* was classified into two sections i.e. *Gnemonomorphi* and *Cylindrostachys* in the monograph^7^. Morphological observation of male strobili has been conducted among different lineages of *Gnetum* e.g. African species^8,9^, Asian arboresent species^3,5^, and Asian lianoid species^10–12^. These results provide important diagnostic characters to further delimit species among different lineages of this genus. As being dioecious, the majority of *Gnetum* species are entemophilous or ambophilous^6,13^. At anthesis, sterile ovules of *G. gnemon* L. and *G. cuspidatum* Blume not only produce sugary pollination drops with rotten scents to attract insects, but also provide nutrition to pollinators as a reward^14,15^.

Despite of these previous efforts, some issues related to sexual reproduction and evolution about *Gnetum* male strobili remain unclear. First, previous studies shows that MADS-box genes are essential in organ identity and subsequent development of female and male strobili in *G. gnemon*^16–18^. However, roles of other genes, e.g. Type I and *TM8* genes in the sexual determination of male strobili have not been investigated. Second, photosynthetic capacity of *Gnetum* leaves have been tested to be low^19,20^, a male strobilus might undergo the improvement of photosynthesis to provide additional resource for the entire course of male strobilus development, but the validation of this statement requires robust evidence. Third, at anthesis, a male strobilus produces sugary pollination drops, the underlying mechanisms that generate the main components in a pollination drop remain poorly understood. Last but not the least, a male strobilus might apply an efficient mechanism to protect against external fungi, bacteria and pathogen, since their ovules are exposed to the surrounding area like other gymnosperms. Molecular mechanisms, again, have not been carefully investigated.

LncRNAs (long non-coding RNAs) take part in transcriptional and post-transcriptional gene regulation in almost all eukaryotic organisms^21–23^, it might control the expression of genes involved in male strobilus development of *G. luofuense*. The roles of lncRNAs might differ across different development stage of male strobili but their potential roles in the regulation of functional genes, so far, have rarely been investigated. It is probably because the RNA-seq based on Illumina platforms produce short reads, preventing us from the assembly of full-length transcrptome. With the advent of third generation sequencing technology, Oxford Nanopore Technologies (ONT) can now be used to sequence complete full-length cDNAs^24^. To date, Nanopore sequencing has been widely used in plant genome sequencing)^25,26^, but rarely in full-length transcript sequencing and investigation of lncRNAs. A recent study has shown that ONT technology generates better quality raw data and provides more accurate data at transcription level than PacBio technology^27^. In the present study, we generated nine full-length transcriptomes from three developmental stages of *G. luofuense* male strobili using Nanopore sequencing technology. Differentially expressed lncRNAs, transcripts and TFs were investigated across the three developmental stages. Our efforts will be made to understand the molecular mechanisms that drive organ evolution and pollination biology in *Gnetum*.

## Results

### Assembly and functional annotation of full-length reads

In the present study, we defined three developmental stages of *G. luofuense* male strobilus—FA represents 1–10 days’ growth, FB represents 10–15 days’ growth, and FC represents 15–25 days’ growth (Fig. 1). A total of 30,261,170 clean reads were generated by Nanopore sequencing, with mean lengths ranging from 993 (FC03) to 1236 bp (FB02) (Table S1). Among these clean reads, 22,997,187 full length (FL) reads with clear primer sequences at both ends were identified. FL reads accounted for 78.05% (1,971,235; FA02) to 79.64% (1,485,322; FA03) of the clean reads (Table S2). All FL reads were clustered and polished, yielding a total of 257,886 consensus reads, ranging from 22,119 (FB01) to 33,351 (FC03) in individual samples (Table S3). To delete redundant reads, all consensus reads were mapped against the reference genome of *G. luofuense* with the mapping rates ranging from 99.26 to 99.40% (Table S4). After mapping, a total of 132,653 non-redundant full-length reads (nFLs) were obtained, ranging from 12,066 (FB01) to 16,689 (FC03) in individual samples (Table S5). A total of 45,036 transcription were functionally annotated by searching against the NCBI non-redundant protein sequence (NR), Swiss-Prot, Clusters of Orthologous Groups of proteins (KOG/COG), Gene ontology (GO), Protein Family (Pfam) and Kyoto Encyclopedia of Genes and Genomes (KEGG) databases (Table S6).

### Identification of CDS and lncRNAs

**Figure 2:**
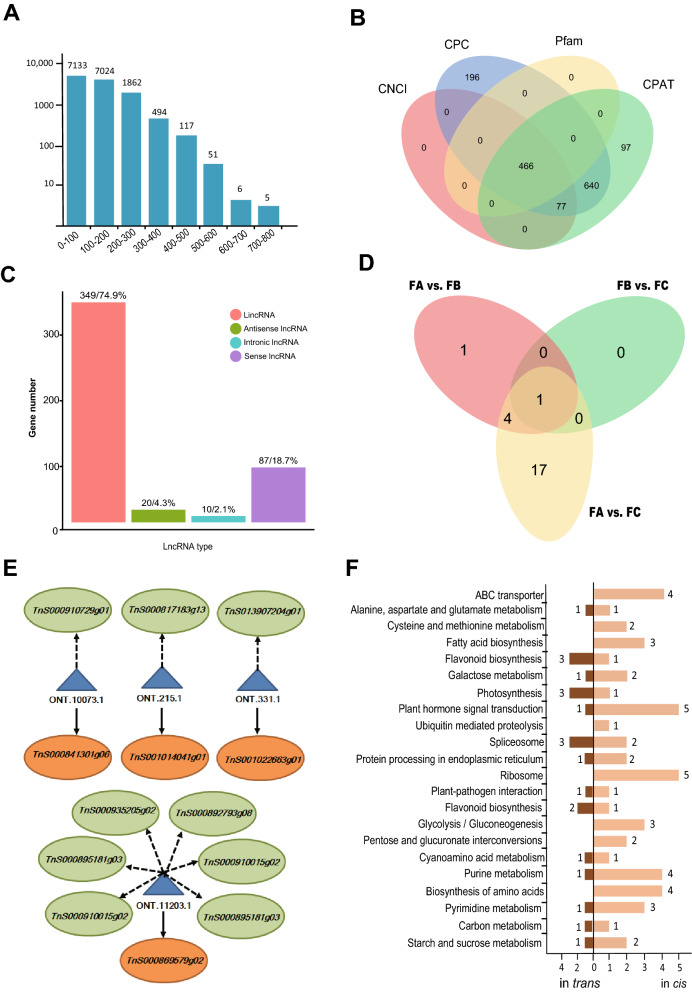
Identification of open reading frames (ORFs) and lncRNAs based on nine full-length transcriptome. (**A**) Length distribution of ORFs detected in all full-length transcripts. (**B**) Venn diagram showing the number of lncRNAs identified using four different approaches: CPC (Coding Potential Calculator), CNCI (Coding-Non-Coding Index), CPAT (Coding Potential Assessment Tool), and Pfam (Protein Family). (**C**) Functional classification and numbers of four lncRNA types. (**D**) Venn diagram showing the overlap in the differential expressed lncRNAs among the three different developmental stages of *G. luofuense* male strobili. (**E**) Representatives of predicted interaction networks among lncRNAs and their target genes. Solid lines and dotted lines represent the expression regulation by the lncRNAs in *cis* and in *trans*, respectively. (**F**) KEGG (Kyoto Encyclopedia of Genes and Genomes) pathway annotations of *cis*- and *trans*-regulated genes regulated by the detected lncRNAs.

We identified a total of 21,720 open reading frames (ORFs), among which 16,692 (76.85%) were complete ORFs with both start and stop codons. The length distribution of the complete ORFs is shown in Fig. 2A. Among the complete ORFs, the average length of the 5′ untranslated regions (UTRs) was 530 bp, and the average length of the 3′ UTR was 557 bp. A total of 15,439 coding sequences (CDS) were detected, with an average length of 394 bp. In addition, 466 lncRNAs with a mean length of 689 nt were identified using four separate methods (Fig. 2B). The lncRNAs comprised 349 lincRNAs (74.9%), 20 antisense lncRNAs (4.3%), 10 intronic lncRNAs (2.1%), and 87 sense lncRNAs (18.7%) (Fig. 2C). The differentially expressed lncRNAs are shown in Fig. 2D, of which we found the number between FA and FC is the largest (22 lncRNAs), while the smallest between FB and FC (one lncRNA). Furthermore, three hundred twenty-six genes were predicted to be regulated by 296 lncRNAs in cis, 218 genes regulated by 81 lncRNAs in trans. Networks of target genes with the regulation of lncRNAs in cis and in trans are shown in Fig. 2E. Moreover, KEGG pathway enrichment analysis was performed for those target genes (Fig. 2F). Our results show that target genes were primarily enriched in “photosynthesis (ko00195)”, “plant-pathogen interaction” (ko04626), “starch and sucrose metabolism” (ko00500), “plant hormone signal transduction” (ko04075), “flavonoid biosynthesis” (ko00941), “amino sugar and nucleotide sugar metabolism” (ko00520, 25).

### WGCNA analysis

**Figure 3:**
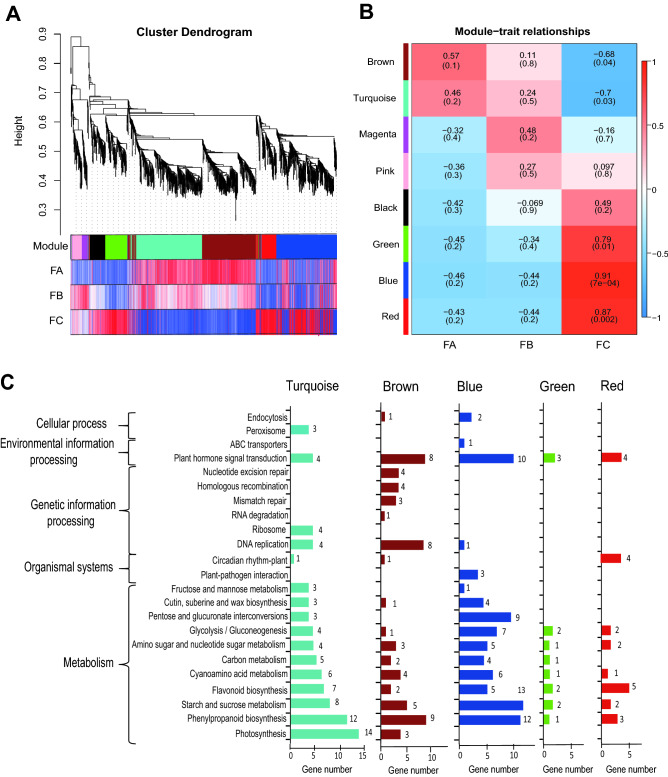
Weighted correlation network analysis (WGCNA) of co-expressed transcripts. (**A**) Hierarchical clustering tree showing co-expression modules based on WGCNA analysis. Each branch in the phylogenetic tree corresponds to an individual gene, and highly interconnected genes are grouped into seven modules. The different colored rows below the phylogeny indicate differentially expressed transcripts in *G. luofuense*. (**B**) Module–trait relationship with physiological indexes. The number represents the correlation coefficient about modules with physiological indexes. The number in the bracket means *p*-value (**C**) KEGG (Kyoto Encyclopedia of Genes and Genomes) pathway annotations of co-expressed genes in the largest five modules.

Expression of all transcripts were quantified with CPM values and listed in Supplementary Dataset File 1. Based on the data, we performed weighted correlation network analysis (WGCNA), and our results show that seven modules of highly correlated TFs across the three developmental stages of *G. luofuense* male strobili (Fig. 3A, B). The three largest modules of enriched transcripts are shown in turquoise genes), brown (349), blue (340). At the FA stage, values of Pearson’s correlation coefficient in the brown and turquoise modules were both positive, but with the q value > 0.05. At the FC stage, however, positive values of correlation coefficient were found among blue, green, and red modules with all *q* values < 0.05. KEGG pathway enrichment analysis was performed for transcripts in the five modules (Fig. 3C). TFs in the turquoise module were primarily enriched in “photosynthesis (ko00195)”, “phenylpropanoid biosynthesis” (ko00940), and transcripts in the brown module were primarily enriched in “phenylpropanoid biosynthesis”, “DNA replication” (ko03030), and “plant hormone signal transduction” (ko04075). Transcripts in the blue module were primarily enriched in “starch and sucrose metabolism”, “phenylpropanoid biosynthesis”, and “plant hormone signal transduction”.

### Detection of DETs

**Figure 4:**
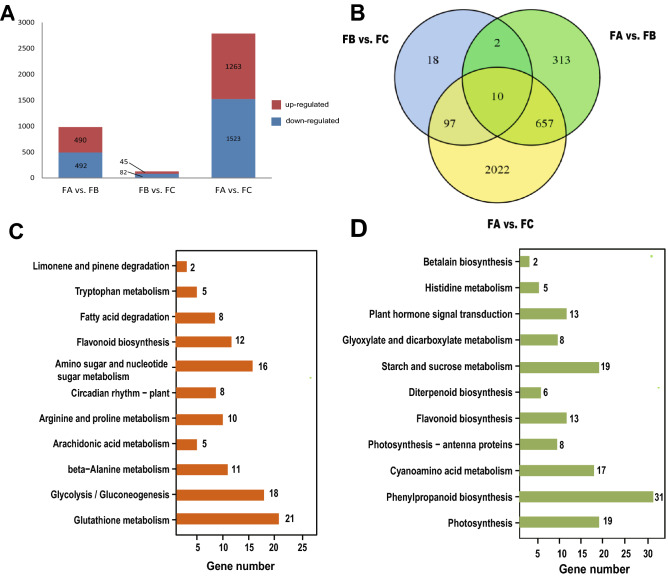
Detected differentially expressed transcripts (DETs) and their annotation. (**A**) Numbers of DETs between different developmental stages of *G. luofuense* male strobili. (**B**) Venn diagram showing the overlap in DETs between different developmental stages of male strobili. (**C**) KEGG (Kyoto Encyclopedia of Genes and Genomes) pathway annotations of up-regulated transcripts between FA and FC. (**D**) KEGG pathway annotation of down-regulated transcripts between FA and FC. (**E**) Two clusters identified based on expression levels of DETs from FA to FC. (**F**) GO enrichment amongst the two clusters with orange boxes denoting significant enrichment of GO annotation.

A total of 3119 differentially expressed transcripts (DETs) were detected (Supplementary Dataset File 2), with the largest number of (2786) between the FA and FC stages; 1263 transcripts were up-regulated and 1523 transcripts were down-regulated in FA versus FC (Fig. 4A). The smallest number of DETs (127) was detected between FB and FC: 45 up-regulated and 86 down-regulated. The number of shared DETs was the largest between FA versus FB and FA versus FC (667), and the number of shared DETs was the smallest between FB versus FC and FA versus FB (12) (Fig. 4B). Among the up-regulated genes, the set of all DETs was significantly enriched in the top three KEGG pathways (Fig. 4C): “glutathione metabolism” (ko00480), “glycolysis /gluconeogenesis” (ko00010), and “amino sugar and nucleotide sugar metabolism” (ko00520). Among the down-regulated genes, the set of all DETs was significantly enriched in the top three KEGG pathways (Fig. 4D): “phenylpropanoid biosynthesis”, “photosynthesis”, “starch and sucrose metabolism” (ko00500).

### TF identification and RT-qPCR validation

**Figure 5:**
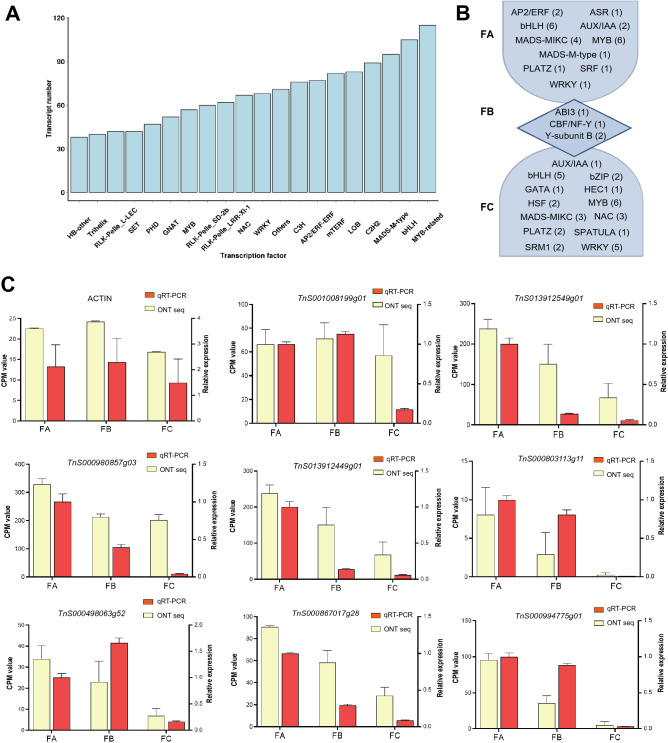
(**A**) A partial list of transcription factors (top 20 gene families) identified in nine full-length transcriptome of *G. luofuense* male strobili. (**B**) Transcription factors highly expressed in FA, FB, and FC with numbers in brackets. (**C**) RT–qPCR of eight genes of interests that encode differentially expressed transcription. Expression of the *ACTIN* gene used as an internal control was also shown. Counts per million (CPM) values from Nanopore sequencing are indicated on the left y-axis, and relative RT-qPCR expression levels are indicated on the right y-axis.

A total of 3138 transcription factors (TFs) was detected, of which MYB-related, MADS-box, and bHLH TFs constituted the most abundant TFs (Fig. 5A, Supplementary Dataset File 3). We further investigated the differentially expressed TFs among the three developmental stages of *G. luofuense* male strobili. We found that bHLH, MYB and MADS-box MIKC were highly expressed at FA and FC, while TFs e.g. Y-subunit B, ABI3 and CBF/NF-Y were highly expressed at FB (Fig. 5B). In addition, we performed RT-qPCR experiments to validate the eight genes of interest, which encode the differentially expressed TFs (Fig. 5C). Results of RT-qPCR are largely congruent with the results of ONT sequencing, but there are exceptions such as genes *TnS001008199g01* and *TnS000980857g03* at the stage of FC and *TnS000498063g52* at the stage of FB. It might because the sampling of RT-qPCR and ONT-sequencing slightly differed at the developmental stages of male strobili.

## Discussion

### Reproductive organ development

MADS-box genes play an essential role in reproductive organ development of seed plants^28–30^. Transcription factors encoded by MADS-box genes consists of two types, i.e. type I (SRF-like) and type II (MEF2-like). A previous study reports that *DEF/GLO*-like gene *GGM15* are differentially expressed during a sterile ovule development of *G. gnemon*^16^. Another study shows that transcription factors (TFs) encoded by *DEF/GLO*-like genes *GGM2* and *GGM15*, *AGL6*-like genes *GGM9* and *GGM11* were examined to form a heterodimer that determine the initiation of male strobilus development in *G. gnemon*^31^. In the present study, four type II genes (including three *TM8-*like genes and one *SQUA*-like gene identified in^32^) as well as one type I genes were differentially expressed across the three developmental stages of *G. luofuense* male strobili (Fig. 5C). These results corroborate that MADS-box genes are important in male strobilus development of *G. luofuense.*

Besides of MADS-box TFs, Aux/IAA TFs are probably also involved in a male strobilus development of *G. luofuense*. Transcriptional factors AUX/IAA are involved in the auxin-response regulation during plant growth^33–35^. In angiosperms, it has been known that Aux/IAA TFs play an important role in gynoecium morphogenesis, ovule development, and formation of primary branch in *Arabidopsis*^36^. Another study shows that *Aux/IAA* genes take effects in reproductive organ development and responses to abiotic stress in rice^37^. In *Gnetum*, a previous study has shown that concentration of endogenous hormones gibberellin A3 (GA_3_) and zeatin riboside (ZR) dramatically increases while indole-3-acetic acid (IAA) declines over the course of male strobilus development in *G. parvifolium*^12^. Another study shows that six Aux/IAA genes are involved in the female strobilus development of *G. luofuense*^38^. In the present study, the results of present study that 13 DETs annotated with hormone and signal transduction are down-regulated between FA and FC (Fig. 4C). Besides, three Aux/IAA TFs were identified and validated to be differentially expressed across the male strobilus development (Fig. 5C). Thus, our results corroborate that plant hormone, e.g. indole-3-acetic acid might play an important role in the male strobilus development of *G. luofuense*.

### Photosynthesis

Photosynthesis in reproductive organs is commonly seen in seed plants. A previous study shows that sepals and petals of tobacco flowers have the photosynthetic capability of fixing CO_2_ and pigment biosynthesis^39^. Another example shows that photosynthetic capacity of grapevine flowers gradually decrease across the procedure of flowering and finally cease at fruit periods^40^. In gymnosperms, female strobili of *Pseudotsuga menziesi* (Douglas-fir) were examined to possess considerable rates of photosynthesis^41^. Another study shows that female strobili of *Pinus sylvestris* (Scots pine) enclosed by aluminum foil yield lower seed weight than the control group^42^, corroborating the presence of photosynthesis in reproductive organs. Our results corroborate the statement that the DETs are annotated in the KEGG pathways e.g. photosynthesis and photosynthesis−antenna proteins (Fig. 4D). For an example, e.g. expression of genes in photosystem I, e.g. *PsaD, PsaE*, and *PsaG* are down-regulated from FA to FC, these genes are regulated by gene differentially expressed lncRNAs (Fig. 2). Accordingly, a scientific question was raised whether reproductive organs of *Gnetum* actually undergo photosynthesis? To address this question, an explicit study of measuring photosynthetic capacity of *G. luofuense* male strobili at different developmental stages is highly desirable in future studies.

### Pollination drop secretion

Pollination drops in general function as a media to capture pollen grains and further transport them to interior nucellus for fertilization^43–45^. *Gnetum* is an entemophilous tropical genus, and a few Asian species (e.g. *G. gnemon*, *G. parvifolium* and *G. luofuense*) are reported to be insect pollinated^13,15,46^. Sugary pollination drops in *Gnetum* are able to attract and provide rewards to pollinators like nectars in angiosperms^43–45^. It has known pollination drops of *Gnetum* are rich in carbohydrates (sucrose, fructose, and glucose), proteins (degradome and secrotome) and amino acids, phosphate, and minerals^6,47^. Our results of WGCNA analysis shows that co-expressed transcripts in the blue module were enriched in KEGG pathways related to carbohydrate metabolism, e.g. starch and sucrose metabolism, glycolysis/gluconeogenesis, and fructose and mannose metabolism (Fig. 3C). These genes were shown to be highly expressed at the late developmental stages of male strobili. For examples, expression of gene *TnS000345359g03* that encodes beta-fructofuranosidase is up-regulated from FA to FC. These results corroborate that *G. luofuense* male strobilus development is closely associated with sugar reproduction; the process might be related to pollination drop production as a response to insect pollinators.

### Defense mechanism

As being reproductive organs, male strobili in *G. luofuense* demands protection against fungi and pathogens throughout their development. An effective manner in *G. luofuense* is to produce a physical barrier—an involucre collar to protect sterile ovules that secrete pollination drops at anthesis. A previous study shows that pollination drops are composed of defense-related proteins, e.g. thaumatin-like proteins, xylosidase, beta-glucodiase, and chitinases secreted from sterile ovules of *G. gnemon* and *G. luofuense*^6,47^. In the present study, we found three genes, i.e. *TnS000958803g05*, *TnS000762467g04*, and *TnS000052095g04* were all up-regulated from FA to FC. Of these genes, genes *TnS000958803g05* and *TnS000762467g04* encode thaumatin-like proteins, which were validated to participate in ovule defense in hybrid yew (*Taxus* × *media*)^48^. The other gene *TnS000052095g04* encodes chitinases, which was considered to be important in protection of female ovules among various species of *Ephedra*^49^ and *Welwitschia*^50^. Thus, this evidence corroborates active defense reaction against external organisms during the male strobilus development of *G. luofuense*.

## Methods

### Plant material sampling

Male strobili of *G. luofuense* were collected from one male individual at the Bamboo Garden at Sun Yat-sen University, Guangzhou, China on April 27, 2019 (voucher No. CH004, the herbarium was identified by Chen Hou and deposited in SYS, Guangzhou, China). Collection of plant samples was permitted by Sun Yat-sen University. Three developmental stages—Stage 1 (1–10 days), Stage 2 (10–15 days), and Stage 3 (15–25 days)—were identified (Fig. 1). Three replicate samples of each developmental stage were prepared from separate male strobili of *G. luofuense*. They were further named as FA (01–03), FB (01–03), and FC (01–03), for a total of nine sequencing samples. Identical samples of *G. luofuense* male strobili were also prepared for RT–qPCR analyses.

### RNA extraction and nanopore sequencing

The collected male strobilus material was snap-frozen in liquid nitrogen and stored at − 20 ℃. A RNeasy Plant Mini Kit (Qiagen, Valencia, CA, USA, product No. 74903) was used to extract total RNA from the nine samples, and relic DNA was removed using RNase-free DNase (Qiagen). The concentration, purity, and integrity of extracted RNA was assessed using 1% agarose gel electrophoresis with NorthernMax gel buffer (Qiagen), a NanoDrop spectrophotometer (ThermoFisher Scientific, Wilmington, DE, USA), and an Agilent 2100 Bioanalyzer (Agilent Technologies, Palo Alto, CA, USA), respectively.

The synthesis of cDNA for Nanopore sequencing was performed according to the protocol from Oxford Nanopore Technologies, UK: 1 μg total RNA was prepared for cDNA libraries using cDNA-PCR Sequencing Kit (SQK-PCS109) protocol provided by Oxford Nanopore Technologies (ONT)^51^. Libraries were then created using a sequencing library preparation kit. We added defined PCR adapters directly to both ends of the first-strand cDNA. The establishment of cDNA libraries was subject to 14 cycles of PCR amplification with LongAmp Taq (NEB). The cycling parameters were set as 94 ℃ for 3 min, followed by 35 cycles of 94 ℃ for 30 s, 56 ℃ for 45 s, 72 ℃ for 1.5 min, and a final extension step of 72 ℃ for 10 min. The PCR products were then subjected to ONT adaptor ligation using T4 DNA ligase (NEB). Agencourt XP beads was used for DNA purification according to ONT protocol. The final cDNA libraries were added to FLO-MIN109 flowcells and libraries were then sequenced using a MinION Mk1B sequencer. Nanopore sequencing data from the nine male strobilus samples were all deposited in the NCBI Sequence Read Archive (SRA) under BioProject accession number PRJNA645614.

### Data processing and genome mapping

Raw sequencing reads were analyzed using MinKNOW version 2.2 (Oxford, UK). Raw reads were filtered with the following settings: read quality score ≥ 7 and read length ≥ 500 bp. Ribosomal RNA was removed by searching against the Silva rRNA database (https://www.arb-silva.de). Full-length reads were identified when primers were detected at both ends of the cleaned reads. All full-length reads were clustered after mapping to the *G. luofuense* reference genome, https://datadryad.org/stash/dataset/10.5061/dryad.0vm3752) using minimap2 version 2.1.7^52^. After each cluster was polished with pinfish (https://github.com/nanoporetech/pinfish), consensus reads were again mapped to the *G. luofuense* reference genome using minimap2. The mapped reads were further collapsed using the cDNA_Cupcake package with a minimum coverage of 85% and a minimum identity of 90%. Consensus reads with sequence differences at the 5′ ends were not considered to be redundant transcripts.

### Functional annotation and classification

All detected genes, including novel genes, were annotated by BLASTX v.2.2.26 searches (E-value < 1 × 10^−5^) of Pfam (http://pfam.xfam.org/), NR (http://www.ncbi.nlm.nih.gov/), and Swiss-Prot (http://www.expasy.org/sprot/) databases, as well as by HMMER v.3.1b2 searches (E-value < 1 × 10^−10^) of the Pfam database^53,54^. In addition, GO enrichment analysis Clusters of Orthologous Groups of Proteins (KOG/COG, http://www.ncbi.nlm.nih.gov/COG/) was performed using the GOseq package implemented in R^55^, and KEGG enrichment analysis was performed using KEGG Orthology^56,57^ based Annotation System using KOBAS^58^.

### Identification of CDS and lncRNAs

We identified CDS of polished non-redundant isoforms using the software TransDecoder version 5.0.2^59^ (https://github.com/TransDecoder/TransDecoder/releases) with a default setting. Prediction of lncRNAs was performed using four methods, the Coding Potential Calculator (CPC)^60^, the Coding-Non-Coding Index (CNCI)^61^, the Coding Potential Assessment Tool (CPAT)^62^, and Pfam. Subsequent to filtering protein-coding reads, lncRNAs were identified as reads that possessed at least 200 nt and two exons. Identified lncRNAs were then classified as either lincRNA, antisense-lncRNA, sense-lncRNA, or intronic-lncRNA. Target genes that are regulated by identified lncRNAs were predicted using the software LncTar (www.cuilab.cn/lnctar)^63^; thus, two types of cis or trans target genes were defined as regulated by lncRNAs in this analysis^64,65^.

### WGCNA analysis

Pearson correlation matrix and network topology analysis were applied to calculate the gene correlation across the nine samples using the R package WGCNA version 1.42^66^ with the following settings: CPM values > 1, fold change > 1, minimum module size of 30, and minimum height for merging modules of 0.055. Then, the adjacency was converted to a topological overlap matrix. A hierarchal clustering tree was constructed using the Dynamic Tree Cut package for R^67^.

### DET detection analysis

Full-length reads were mapped to the *G. luofuense* reference genome mentioned above, and mapped reads with coverage above five were saved. Transcript expression was quantified as counts per million (CPM), where CPM = (reads mapped to transcript/total reads mapped in one sample) × 1,000,000. Differential expression analysis between each pair of developmental stages was performed using DESeq version 1.10.1 (https://bioconductor.org/packages/release/bioc/html/DESeq.html)^68^. The t-test was used to judge the statistical significance of expression difference, and the threshold of *P*-value was determined with the FDR in multiple testing. Three replicates at each developmental stages were set as an independent group for pairwise comparisons. The resulting *P*-values were adjusted using Benjamini and Hochberg’s^69^ approach in order to control the false discovery rate. Genes with FDR-adjusted *p-values* < 0.01 and fold changes ≥ 2 were defined as DETs. KEGG enrichment analyses were performed as described above. Out of these DETs, transcript expression of three replicates at each developmental stage was averaged, and denoted FA, FB, and FC.

### TF identification and RT–qPCR validation

Transcription factors were identified and their genes were assigned to different families using iTAK version 1.7 (http://bioinfo.bti.cornell.edu/tool/itak)^70^. We selected eight differentially expressed TFs for gene expression validation by RT*–*qPCR referred to the MIQE guidelines. Primers were designed using Primer Premier version 5.0^71^, and information on the RT–qPCR protocol is presented in Supplementary Dataset File 1. The cDNA was synthesized from 2 μl of total RNA were extracted from the nine samples using a PrimeScript RT Master Mix (Perfect Real Time) (TaKaRa, China). For each sample, three independent analyses were performed, and the mean and standard deviation of the RT–qPCR gene expression values were calculated. The entire experiment was performed in Applied Biosystems Real-Time PCR Instruments-QuantStudio 6 (ThermoFisher Scientific, China). The amplification program consisted of 2 min of initial denaturation at 95 ℃, followed by 40 cycles of 20 s at 94 ℃, 20 s at 58 ℃, 20 s at 72 ℃. When producing melting curves, the amplification program was set at 30 s at 94 ℃, followed by 30 s at 65 ℃ and 30 s at 94 ℃. The *G. luofuense* actin gene was used as an endogenous control to estimate the relative expression of TFs using the ΔΔCt method^72^. Based on the slope of the standard curve, amplification efficiency was calculated in the website sever (https://www.thermofisher.com/uk/en/home/brands/thermoscientific/molecularbiology/molecular-biology-learning-center/molecular-biology-resource-library/thermoscientific-web-tools/qpcr-efficiency-calculator.html).When amplification efficiency was all close to 100%, relative expression values were calculated as −2∆∆*C*_*t*_, of which ∆∆*C*_*t*_ = ∆*C*_*t*_—the average values of three replicates of ∆*C*_*t*_, and ∆*C*_*t*_ = *C*_*t*_ (genes of our interest) – *C*_*t*_ (actin gene).

## Supplementary Information

Supplementary Information 1.

Supplementary Information 2.

Supplementary Information 3.

Supplementary Information 4.

Supplementary Information 5.
