# Supplementary material for: Revealing the developmental dynamics in male strobilus transcriptome of Gnetum luofuense using nanopore sequencing technology
Source: Sci Rep. 2021 May 18;11:10516. doi: 10.1038/s41598-021-90082-0 (PMC8131605; doi:10.1038/s41598-021-90082-0)
Supplement: Supplementary file 1 — Supplementary Information 1. [file 41598_2021_90082_MOESM1_ESM.docx]

**Revealing the developmental dynamics in male strobilus transcriptome of *Gnetum luofuense* using Nanopore sequencing technology**

**Chen Hou^1,2^, Yuxin Tian^3,4^, Yingli Wang^1,2^, Huiming Lian^1,2^, Dongcheng Liang^1,2^, Shengqing Shi^5^, Nan Deng^3,4^*, Boxiang He^1,2^***

1. Guangdong Provincial Key Laboratory of Silviculture, Protection and Utilization, Guangdong Academy of Forestry, Guangzhou, 510520, China

2. Guangdong Academy of Forestry, Guangzhou, Guangshanyilu No. 233, Longdong District, 510520, China 510520, China

3. Hunan Academy of Forestry, Changsha, Hunan, No. 658 Shaoshan Road, Tianxin District, Changsha, 410004, China

4. Hunan Cili Forest Ecosystem State Research Station, Cili, Hunan, Changsha, 410004, China

5. Research Institute of Forest Ecology, Environment and Protection, Key Laboratory of Forest Ecology and Environment of State Forestry Administration, Chinese Academy of Forestry, 1958 Box, Beijing 100091, China*

Correspondence and requests for materials should be addressed to B.H. and N.D. (emails: heboxiang@163.com; idengnan@me.com)
