# Supplementary material for: Revealing the developmental dynamics in male strobilus transcriptome of Gnetum luofuense using nanopore sequencing technology
Source: Sci Rep. 2021 May 18;11:10516. doi: 10.1038/s41598-021-90082-0 (PMC8131605; doi:10.1038/s41598-021-90082-0)
Supplement: Supplementary file 2 — Supplementary Information 2. [file 41598_2021_90082_MOESM2_ESM.pdf]

Table S1 Statistics of clean reads generated by Nanopore sequencing

Sample name	Number of raw reads	Number of base pairs (bp)	N50	Mean length (bp)	Maximum length (bp)
FA01	2,298,569	2,559,418,749	1229	1113	8342
FA02	1,978,082	2,286,372,991	1287	1155	9114
FA03	1,872,243	2,184,396,831	1299	1166	9664
FB01	1,748,837	2,007,288,649	1285	1147	8154
FB02	3,220,124	3,981,476,718	1386	1236	10,662
FB03	3,519,688	4,039,394,138	1278	1147	10,326
FC01	2,081,078	2,448,636,465	1314	1176	9085
FC02	2,488,157	2,881,186,726	1294	1157	8927
FC03	3,790,409	3,766,792,826	1059	993	8512

Table S2 Numbers and percentages of full-length reads

Sample name	Number of clean reads (except rRNA)	Number of full-length reads	Full-length percentage (FL%)
FA01	2,291,165	1,795,096	78.35%
FA02	1,971,235	1,538,606	78.05%
FA03	1,865,129	1,485,322	79.64%
FB01	1,742,602	1,379,367	79.16%
FB02	3,209,051	2,516,007	78.40%
FB03	3,507,675	2,792,687	79.62%
FC01	2,074,495	1,631,419	78.64%
FC02	2,479,358	1,945,619	78.47%
FC03	3,776,421	2,959,056	78.36%

Table S3 Statistics of consensus reads

Sample name	Number of consensus reads	Number of base pairs (bp)	N50	Mean length (bp)	Maximum length (bp)
FA01	27,109	31,554,920	1348	1164	4429
FA02	23,426	28,314,236	1394	1208	4489
FA03	23,816	28,639,915	1391	1202	4380
FB01	22,119	26,391,827	1373	1193	4179
FB02	35,684	47,367,607	1548	1327	5978
FB03	37,927	45,699,232	1406	1204	4844
FC01	25,698	31,518,631	1420	1226	5002
FC02	28,756	34,938,723	1418	1215	4290
FC03	33,351	34,192,552	1192	1025	2516

Table S4 Statistics of mapped clean reads against the *G. luofuense* genome

Sample	Total Reads	Mapped reads	Mapped rates %
FA01	3,507,675	3,484,920	99.35%
FA02	1,971,235	1,958,797	99.37%
FA03	1,865,129	1,852,304	99.31%
FB01	1,742,602	1,731,379	99.36%
FB02	3,209,051	3,189,820	99.40%
FB03	2,291,165	2,275,544	99.32%
FC01	2,074,495	2,060,940	99.35%
FC02	2,479,358	2,461,012	99.26%
FC03	3,776,421	3,748,365	99.26%

Table S5 Statistics of non-redundant consensus reads

Sample name	Number of non-redundant reads	Number of base pairs (bp)	N50	Mean length (bp)	Maximum length (bp)
FA01	13,886	18,004,781	1476	1296	4429
FA02	12,601	16,666,178	1501	1322	4489
FA03	12,814	17,030,636	1514	1329	4380
FB01	12,066	15,676,639	1476	1299	4179
FB02	18,229	26,297,686	1661	1442	5978
FB03	18,006	24,247,013	1546	1346	4844
FC01	13,549	18,235,991	1540	1345	5002
FC02	14,813	19,782,427	1533	1335	4290
FC03	16,689	18,600,729	1277	1114	2516

Table S6. Summary of annotated non-redundant FL transcripts by the seven databases

Databases	Novel gene numbers
COG	16,538
GO	25,621
KEGG	20,821
KOG	26,974
NR	44,326
Pfam	32,735
Swissprot	34,318
Total annotated genes	45,036

Table S7. Information of primers and RT-qPCR systems

ID	Sequence(5'- 3')	Product Length(bp)
Actin.F	TTGTAGGTCGCCCTCGTC	
Actin.R	CTCCCTGTTAGCCTTTGG	
TnS013912549g01.F	CTTCACGAGTTCTCCAACCC	140
TnS013912549g01.R	TCAATCCGACCATGCAACTT	
TnS013912449g01.F	TACGAGCTTTCGGTTCTGTG	160
TnS013912449g01.R	GTTGACTTTCCTGGGCATCA	
TnS000767679g01.F	AGAAGAGGAGGATGAGCGTT	111
TnS000767679g01.R	AGGCTCGCAATTCTGTGATT	
TnS000803113g11.F	ACAACAACAGCTCGTCCATT	171
TnS000803113g11.R	TCAACATAAGCCTGCGACTC	
TnS000889809g02.F	GTGACTGCTACTGCTTCTCC	193
TnS000889809g02.R	CAGAGCTAGACGACTCCTCA	
TnS000976213g02.F	GGAGCTCGACCGTGAATATC	205
TnS000976213g02.R	TTCAATCTGAGCAGCAGACC	
TnS000867017g28.F	TTACCGGAAACTCGCAGAAG	157
TnS000867017g28.R	CTCACGAACATCTCCCAAGG	
TnS000994775g01.F	CAGCAGCGACTTCTACTCTC	163
TnS000994775g01.R	AGAGCGTTTGGTTCCTGAAA	

RT-qPCR systems

Bestar® SybrGreen qPCRmasterMix	5 μl
PCR Forward Primer（10μM）	0.25μl
PCR Reverse Primer（10μM）	0.25μl
cDNA	2μl
ddH_2O	2.5μl
Bestar® SybrGreen qPCRmasterMix	5 μl
